# Overweight in youth and sleep quality: is there a link?

**DOI:** 10.1590/2359-3997000000265

**Published:** 2017-06-14

**Authors:** Susana Rebelo Pacheco, Ana Margalha Miranda, Raquel Coelho, Ana Cristina Monteiro, Graciete Bragança, Helena Cristina Loureiro

**Affiliations:** 1 Pediatric Department Hospital Prof. Doutor Fernando Fonseca Amadora Portugal Pediatric Department, Hospital Prof. Doutor Fernando Fonseca, Amadora, Portugal

**Keywords:** Children, obesity, sleep quality

## Abstract

**Objective:**

Overweight seems to be related to a higher prevalence of sleep disturbances. Decreased sleep duration and altered sleep quality are risk factors for obesity. Our aim was to compare the sleep pattern of overweight children with that of a matched control group and assess the relationship between sleep quality and obesity.

**Materials and methods:**

Retrospective cohort study comparing 41 overweight children with a normal-weight control group, both submitted to polysomnography. The samples were matched for age, sex, and apnea-hypopnea index. Body mass index (BMI) z-scores were calculated using World Health Organization (WHO) growth charts. Insulin resistance in the study group was determined using the homeostatic model assessment for insulin resistance (HOMA-IR). Sleep patterns were compared. The statistical analysis was performed using SPSS^®^ version 21.

**Results:**

The mean age (± standard deviation) of the population was 10 ± 3.4 years (min. 5 years; max. 17 years). Fifty-six percent of the participants in both groups were girls. N3% was lower in the study group (18.95 ± 6.18%) compared with the control group (21.61 ± 7.39%; t (40) = 2.156, p = 0.037). We found a correlation in the study group between HOMA-IR and N3% (Rs = -0.434, p = 0.008).

**Conclusion:**

The present study suggests a link between overweight/obesity and altered sleep quality due to compromised non-rapid eye movement sleep, an indirect marker of sleep quality. There was also a link between slow-wave sleep duration and insulin resistance. We must find a strategy to provide adequate slow-wave sleep duration to reduce the obesity epidemic at young ages. Further research is needed.

## INTRODUCTION

The prevalence of childhood overweight and obesity has significantly increased in the past decades, representing a global epidemic and a major public health concern (1). Childhood obesity contributes to significant physical, psychological, and economic burden. It often continues into adulthood and confers a major risk for insulin resistance, impaired glucose tolerance, hypertension, dyslipidemia, and cardiovascular disease (2).

Attempted interventions to improve diet and physical activity patterns among adolescents have had limited success at controlling pediatric obesity (3). Identification of early childhood modifiable risk factors should be a priority (2,3).

While the prevalence of obesity has increased, lifestyle changes have led to a decrease in night sleep hours among children and adolescents due to later bedtimes with unchanged rise times. Worldwide studies estimate that 20%-30% of children and 6%-37% of adolescents report problems related to prolonged sleep latency, difficulty initiating and maintaining sleep, frequent nocturnal awakenings, and poor sleep quality accompanied by significant daytime impairments (4,5).

Short sleep has been identified as a risk factor for obesity in children and adults, and several studies have reported a strong relationship between sleep duration and higher BMI (2). However, it has been difficult to draw immediate conclusions on the consistency of the association, direction of causality, and likely mechanisms involved (6).

The underlying pathogenic pathways are complex and unresolved, but it is known that sleep, specially slow-wave sleep (SWS), has a major role in body restorative processes and energy metabolism. Poor sleep might impact glucose regulation, inflammatory markers, and appetite regulating hormones, promoting food intake (7-9). Although a strong relationship between sleep deprivation, increased food intake, and obesity has been documented, only a small number of studies have yet examined how eating behavior may be related to sleep quality and quantity (9,10). It has been demonstrated that reduced subjective sleep quality scores are associated with behaviors like binge eating and emotional eating (11,12).

Other pathogenic mechanisms seem to play a role in the relationship between sleep deprivation and obesity. The autonomic nervous system is an important contributor in coordinating energy homeostasis and plays a role in the pathophysiology of obesity. Sympathovagal imbalance, a marker of autonomic dysfunction, is present in obese children with sympathetic hyperactivity and decreased parasympathetic functioning when compared with normal-weight children (12,13). Autonomic dysfunction has been linked to altered sleep duration, sleep fragmentation, sleep disorders like insomnia, and subjective/objective reports of poor sleep quality (14,15).

Cross-sectional studies reveal that shorter sleep duration, poor sleep quality, sleep disturbances, and a delayed sleep phase pattern are associated with larger body composition, greater central adiposity, and increased obesity rates among adults and youths (4,14,16). In addition to these potential consequences, short sleep may contribute to increased risk of cognitive and behavioral problems (17).

The aim of the present study was to compare the sleep pattern of overweight children with a matched control group and assess the relationship between sleep quality and overweight/obesity.

## MATERIALS AND METHODS

### Study type

Retrospective cohort study comparing 41 overweight and obese children (study group) to 41 non-overweight children (control group).

### Study population

The study population included children and adolescents submitted to a sleep study. Children were referred to a sleep specialist to exclude sleep disturbances in the context of snoring. Overweight and obese children were selected to make up the study group. The control group was randomly selected from non-overweight patients. Both groups were matched for age, sex, and apnea-hypopnea index (AHI).

### Measures

#### Anthropometrics

Children were weighed (to 0.1 kg) with a calibrated scale, and their heights were measured (to 0.1 cm) with a stadiometer at the first sleep appointment by the same observer using the same equipment. The International System of Units was used. Weight was measured in kilograms (kg) and height in centimeters (cm). BMI values were calculated dividing weight (kg) by squared height (m^2^). Age-sex-adjusted BMI standard deviation scores (z-scores) were computed and distributed using World Health Organization (WHO) growth charts and the official WHO z-score calculator. Overweight was defined as a BMI z-score > 1 and obesity as a BMI z-score > 2. Normal weight was defined as a BMI z-score ≤ 1. These definitions were based on WHO recommendations (18).

#### Sleep data by polysomnography

All children underwent nocturnal type 1 polysomnography (PSG) at our sleep laboratory during an acute illness free period. All PSG were performed using electroencephalography (frontal, central, and occipital channels), chin electromyography, two leg channels, electrooculogram, piezo-electric chest and abdominal belts, simultaneous pressure transducer and thermistor for nasal flow, transcutaneous (Tc) carbon dioxide and oxygen saturation monitoring, snore microphone, electrocardiography, and simultaneous video monitoring. All PSG results were scored by the same professional, adequately qualified in PSG analysis (with a Master degree in sleep sciences).

Detailed sleep parameters were measured, including total sleep time (TST); wake after sleep onset (WASO); non-rapid eye movement sleep (NREM) divided into three stages: stages 1 (N1) and 2 (N2), light sleep stages, and stage 3 (N3), SWS; rapid eye movement sleep (REM) latency; sleep efficiency; REM; SWS percentage; snoring percentage; number of arousals per hour; AHI and oxygen saturation nadir. Periodic leg movements (PLM) and PLM arousal index, and parasomnias were also investigated. Sleep architecture was scored using the 2012 American Academy of Sleep Medicine guidelines (AASM 2012) (19). The following definitions were used: 1. Obstructive sleep apnea was defined as the cessation of airflow for two respiratory cycles; 2. Hypopnea was defined as a decreased in amplitude of the airflow signal by 50% lasting two respiratory cycles and accompanied by an oxygen desaturation > 3%; 3. An AHI > 1/hr was considered as a diagnosis of obstructive sleep apnea syndrome (OSAS); 4. Habitual snoring was defined as snoring not accompanied by oxygen desaturation, arousals or changes in air flow; 5. Periodic limb movements of sleep were defined as repetitive jerking of the legs during sleep of more than five movements per hour, 0.5–5 sec in duration, in clusters of four or more separated by 5–90 sec intervals; 6. Parasomnias were defined by the occurrence of confused arousal, sleepwalking episodes, sleep terrors, and nightmare disorders according to the International Classification of Sleep Disorders (ICSD), 2^nd^ edition; 7. TST corresponded to the sum of total sleep during stages N1, N2, N3, and REM; 8. WASO encompassed the time spent awake after sleep had been initiated and before final awakening; 9. Sleep efficiency was calculated using the ratio of total minutes of sleep (N1 + N2 + N3 + REM) and time spent in bed; 10. Sleep latency was defined by the time, in minutes, elapsed from lights out to sleep onset; 11. REM latency represented the time from the first epoch of sleep to the first REM sleep.

The normal ranges of sleep variables are presented in Table 1.

**Table 1:** Normal values of sleep pattern on polysomnography (adapted from the American Academy of Sleep Medicine guidelines) and comparison of sleep patterns between the control and study groups

	Normal values	Control group		Study group		Comparison p*
	
		Mean	SD	Mean	SD	
Duration (reported, h)	-	9.43	1.08	9.26	1.45	0.574
TST (min)	> 330	416.41	55.92	426.95	61.62	0.353
WASO (min)	-	61.40	48.64	67.07	47.25	0.354
Latency (min)	< 30	25.42	26.14	21.07	31.40	0.091
Efficiency (%)	> 85 TST	82.95	10.66	82.89	11.84	0.892
AHI (nr/h)	< 1	3.00	2.30	2.85	2.01	0.199
Arousals (nr/h TST)	< 14	12.27	5.17	12.85	5.82	0.444
N1 (%)	0-5	2.98	2.69	3.38	2.33	0.310
N2 (%)	< 55	61.77	8.50	63.95	6.22	0.144
N3 (%)	15-20	21.61	7.39	18.95	6.18	**0.037**
REM (%)	20-25	13.62	5.47	13.25	4.32	0.726
REM sleep latency (min)	< 120	157.95	74.01	161.57	59.03	0.688
Mean SpO_2_ (%)	> 95	95.54	2.19	95.59	1.36	-
Min. SpO_2_ (%)	> 94	93.12	3.14	92.41	3.41	-

TST: total sleep time; WASO: wake after sleep onset; AHI: apnea-hypopnea index; N1: stage 1 of non-rapid eye movement sleep; N2: stage 2 of non-rapid eye movement sleep; N3: stage 3 of non-rapid eye movement sleep; REM: rapid eye movement sleep; SpO_2_: peripheral capillary oxygen saturation; SD: standard deviation.

* Statistical comparison of the sleep pattern in the control and study groups.

Weekday sleep duration was calculated using parents or self-reported usual weekday bedtime and wake time.

#### Evaluation of insulin resistance using the HOMA-IR

In the study group, night fasting plasma glucose and insulin levels were measured in our hospital laboratory. Parental consent was obtained for blood sampling at the Endocrinology appointment. No laboratory results from the control group were available, as these are not regular laboratory studies required at a sleep appointment.

We evaluated insulin resistance using the homeostasis model assessment for insulin resistance (HOMA-IR). HOMA-IR was calculated with the equation: HOMA-IR = fasting insulin (µU/mL) x fasting glucose (mg/dL) / 405. The cutoff level to determine insulin resistance was 3.16 (20).

## Statistical analyses

Descriptive statistics were computed for all variables. Sleep patterns were compared using Student’s *t* test for paired samples and Wilcoxon test. We used Spearman’s correlation to correlate the sleep pattern and the HOMA-IR index. All data were analyzed with IBM SPSS Statistics 21^®^ software (SPSS, Inc., Chicago, IL). We defined a p value of < 0.05 to indicate statistical significance.

## RESULTS

### Descriptive statistics

Descriptive data are presented as a percentage for discrete variables and as mean (± standard deviation [SD]) for continuous variables.

Each group (study and control) comprised 41 subjects matched for gender, age, and AHI, who were evaluated with PSG. The age of the participants ranged from 5 to 17 years (mean 10.95 ± 3.4 years), and girls represented 56% of the sample. The mean BMI z-score of our control and study groups were -0.25 ± 0.80 and 2.74 ± 0.56, respectively. Our study group was mostly composed of obese children (90.2% with BMI z-scores > 2; 9.6% with BMI z-scores > 1 and < 2).

Means and SDs for measures of sleep are presented in Table 1. The mean sleep duration was similar in the control and study groups (9.43 h vs. 9.26 h). Mean latency was 25.42 and 21.07 minutes in each group, respectively. Sleep latency lasted more than 30 minutes in 17% of the study group (7 children) and in 24.4% of the control group (10 children). Sleep efficiency was < 85% of the TST in 18 study group children (43.9%) and 20 control group children (48.8%). SWS proportion was under 15% in 12 (29.27%) study cases (control group had 10 children – 24.3%) and was higher than 20% in 18 (43.9%) study cases (control group had 24 cases – 58.5%). In 97.6% cases in the study group, the REM proportion was < 20% (90.2% in the control group). In 15 study group children (36.6%) and 14 control group ones (41.1%), counted arousals were > 14 per hour during the PSG. Sleep efficiency was reduced in both groups (study group 82.89 ± 11.84%; control group 82.95 ± 10.66%). The remainder sleep constants were normal in both groups.

In the study group, the mean HOMA-IR was 2.38 ± 1.54, and seven cases fulfilled the criteria for insulin resistance.

### Comparative statistics

The SWS duration (N3) was lower in our study group (study group: 18.95 ± 6.18%; control group: 21.61 ± 7.39%). A statistically significant difference was found comparing SWS in both groups (p = 0.037). No statistical differences were found comparing other sleep constants (p > 0.05). The comparison in sleep patterns between the control and study groups is presented in Table 1.

We found a moderately negative correlation (Rs = -0.434) between HOMA-IR and %N3 (p = 0.008). Only a low positive correlation was found between HOMA-IR and %REM (Rs = 0.348, p = 0.037) and number of arousals (Rs = 0.366, p = 0.028). No other statistically different correlations were found comparing HOMA-IR and sleep variables. The correlation between sleep pattern and HOMA-IR in the study group is presented in Table 2.

**Table 2:** Correlation between sleep pattern and HOMA-IR in the study group

Study group		
	Correlation	p
TST (min)	0.014	0.933
WASO (min)	-0.126	0.463
Latency (min)	-0.027	0.875
Efficiency (%)	0.102	0.553
Arousals (nr/h)	**0.366**	**0.028**
N1 (%)	0.106	0.540
N2 (%)	0.000	0.999
N3 (%)	**-0.434**	**0.008**
REM (%)	**0.348**	**0.037**
REM sleep latency (min)	0.115	0.505
Mean SpO_2_	-0.201	0.239
Min. SpO_2_	-0.1	0.562

TST: total sleep time; WASO: wake after sleep onset; AHI: apnea-hypopnea index; N1: stage 1 of non-rapid eye movement sleep; N2: stage 2 of non-rapid eye movement sleep; N3: stage 3 of non-rapid eye movement sleep; REM: rapid eye movement sleep; SpO_2_: peripheral capillary oxygen saturation; SD: standard deviation.

## DISCUSSION

The increasing prevalence of overweight and obesity in children and youth is a major public health concern, as it is linked to multiple chronic diseases and premature mortality. Interventions to improve lifestyle have been applied and efforts have been made to understand the different pathogeneses of overweight and obesity (1-3,21).

Growing scientific evidence identifies disturbed sleep as a risk factor for obesity in children. In fact, duration of sleep and sleep quality seem to be related to obesity (22-25). Poor sleep has also been associated with increased waist circumference, an index of central adiposity (7,15).

Most studies used data collected from self-reporting questionnaires or sleep diaries and described sleep duration without an objective sleep quality analysis.

In our study, the mean sleep duration was similar in both groups, with no significant difference found between them (9.43 h vs. 9.26 h, p = 0.574), although other published studies have found that obese children usually sleep less than normal-weight ones (2,4). Sleep duration was the only self-given subjective data. As known from the literature, sleep reports are found to over-report sleep duration. Parents and children were directly asked about the time spent in bed during weekdays. The self-reported time could be a reason for not finding a significant short sleep duration difference between the overweight/obese children and the normal-weight children.

In our study, TST was short in both groups, but no significant difference was found between the groups. This may be due to the PSG being performed in a hospital room where the first night effect must be taken into account. Also, the PSG was performed during a weekday. Spruyt and cols*.* found no significant difference in daily TST but described a shorter TST duration on weekends in obese children (p = 0.03) (24).

When analyzing the PSG data from both groups, we noticed that sleep efficiency was reduced in both (study group 82.89 ± 11.84%; control group 82.95 ± 10.66%). This may be related to the first night effect and a shorter sleep duration. We found no significant difference in the number of arousals, a fact that could also explain a reduced efficiency.

All other sleep constants were normal in both groups, with the exception of N3%. The study group had a decreased percentage of SWS, and a statistically significant difference was found comparing the SWS results in both groups (18.95 ± 6.18% vs. 21.61 ± 7.39%; p = 0.037). SWS is thought to play an important role in cerebral restoration and recovery, and is involved in maintenance and consolidation of sleep (26). Being the most important sleep phase in regulating metabolism, selective deprivation of SWS is related to insulin metabolism deregulation, increased cortisol secretion, and decreased growth hormone concentration. Consequently, reduced SWS and sleep fragmentation result in disturbed sleep quality and may be related to weight gain and z-score elevation due to an important change in body metabolism (22,26).

The literature provides increasing support for the pathophysiology of sympathovagal imbalance having a contributing role in the association between sleep pattern and obesity. SWS has predominantly parasympathetic and reduced sympathetic activity. Sleep disturbances are associated with higher nocturnal arousals, which enhance sympathetic activity leading to increases in urinary catecholamine levels, heart rate, blood pressure, and sympathovagal imbalance among adults and youth (6,13). Although this was not observed in our study, maybe due to the small study sample, it is known that obese children have a higher sympathovagal imbalance (15).

Shortened sleep is reported to support obesogenic behaviors with lower levels of physical activity, higher food intake, and selection for junk food and a carbohydrate-rich diet (9,14,27). This seems to be caused by stimulation of neurons of the ventral tegmental area related to the reward sensation after food intake (28).

On the other hand, sympathetic stimulation that occurs with sleep deprivation might contribute to metabolic deregulation (29).

It is well known in the literature that neurobehavioral changes associated with puberty have a significant influence on sleep organization. The main changes include a delayed sleep phase, which involves a tendency for later bedtimes and rise times; shorter sleep, which is associated with increased levels of daytime sleepiness; and irregular sleep patterns, which involve sleeping very little on weekdays and sleeping longer during weekends to partially compensate for this sleep loss (5,17). However, due to the modest size of the sample and no access to the participant’s Tanner stages, these factors were not taken into account during the statistical analyses in the present study.

The mean HOMA-IR in the study group was normal (2.38 ± 1.54), but seven cases fulfilled the criteria for insulin resistance (HOMA-IR > 3.16). Besides, we found a moderately negative correlation (Rs = -0.434) between HOMA-IR and %N3 (p = 0.008). These data seem to reinforce the already described relation between reduced SWS and dysfunctional metabolism. We found no relation between HOMA-IR and TST and efficiency, as mentioned in previous articles (30).

Puberty is associated with transient insulin resistance, which normally resolves by the end of this period. Girls are usually more insulin resistant at all Tanner stages than boys (30). This was not taken into account in the present study due to the limitations cited above (small sample size and absent data on Tanner stages).

We were unable to find other matching published data, mostly because the majority of the studies used exclusively self-reported questionnaires to evaluate sleep quality and no qualitative PSG data.

Our study has a number of strengths. First, we used an objective methodology to assess sleep quality using overnight PSG in all patients and no self-reported questionnaire on the perception of sleep quality. This is an important fact, as the majority of published studies do not use these objective data. Second, the study and control group were matched for age, sex, and AHI, limiting confounders. Third, no self-reported height or weight data were used, and BMI was calculated using objective values.

The study has some limitations. The sample size (41 children in each group) may be a major limitation. Although we used objective PSG data, the total weekly sleep duration was self-reported, as already described above. Although both groups were matched, other confounders could exist: ethnic, sexual maturity, chronic illness, socioeconomic status, the frequency of snacking and other health-related behaviors or nutritional habits. Another limitation may be that all the studies were performed at the hospital during a single night period; therefore, the first night effect must be taken into account. The PSG data were validated for the age of the population, making the comparison possible.

Still, many questions need answers to determine causality and exact changes in sleep pattern.

In conclusion, the present study found a preliminary support of altered sleep quality in overweight and obese children compared with normal-weight ones, with an impact on insulin sensitivity and glucose metabolism. This matches previously published information. A link seems to exist between overweight/obesity and altered SWS duration, an indirect marker of sleep quality, related to its metabolic and hormonal homeostatic function. Strategies providing behavioral sleep interventions and adequate SWS duration are a fundamental objective to be pursued in order to reduce the obesity epidemic at young ages. Adequate sleep duration and good sleep quality seem to be low-cost approaches to ensure homeostasis and help control increased weight among our youth. There is still need for further research evaluating the sleep pattern of overweight children and youth, analyzing possible confounding factors in larger populations.

Acknowledgments: there were no contributions to the article from individuals other than the authors.

Disclosure: no potential conflict of interest relevant to this article was reported.
